# Engineering Immune Cells for *in vivo* Secretion of Tumor-Specific T Cell-Redirecting Bispecific Antibodies

**DOI:** 10.3389/fimmu.2020.01792

**Published:** 2020-08-13

**Authors:** Belén Blanco, Ángel Ramírez-Fernández, Luis Alvarez-Vallina

**Affiliations:** ^1^Cancer Immunotherapy Unit (UNICA), Department of Immunology, Hospital Universitario 12 de Octubre, Madrid, Spain; ^2^Immuno-Oncology and Immunotherapy Group, Instituto de Investigación Sanitaria 12 de Octubre (imas12), Madrid, Spain

**Keywords:** cancer immunotherapy, T cell-redirection, bispecific antibodies, chimeric antigen receptors, *in situ* secretion

## Abstract

Immunotherapeutic approaches based on the redirection of T cell activity toward tumor cells are actively being investigated. The impressive clinical success of the continuously intravenously infused T cell-redirecting bispecific antibody (T-bsAb) blinatumomab (anti-CD19 x anti-CD3), and of engineered T cells expressing anti-CD19 chimeric antigen receptors (CAR-T cells) in hematological malignancies, has led to renewed interest in a novel cancer immunotherapy strategy that combines features of antibody- and cell-based therapies. This emerging approach is based on the endogenous secretion of T-bsAbs by engineered T cells (STAb-T cells). Adoptive transfer of genetically modified STAb-T cells has demonstrated potent anti-tumor activity in both solid tumor and hematologic preclinical xenograft models. We review here the potential benefits of the STAb-T strategy over similar approaches currently being used in clinic, and we discuss the potential combination of this promising strategy with the well-established CAR-T cell approach.

## Introduction

The immune system plays an important role in shaping the immunogenicity of tumors (1). The T cell receptor (TCR)-mediated recognition of processed tumor-associated antigens (TAAs) drives the elimination or sculpting of developing cancer cells, which can generate immune-resistant cell variants (1, 2). Due to this selective immune pressure, these variant cells display a multitude of evasion mechanisms from immune recognition and destruction, such as abnormalities in the antigen presentation machinery (2), and the generation of an immunosuppressive environment that promotes tumor growth (3). In the past few decades extensive research has been made to develop cancer immunotherapy approaches aimed at stimulating anti-tumor T cell responses (4, 5). Most notably the emergence of immune checkpoint inhibitors blocking negative regulators of T cell immunity (6), the systemic administration of bispecific antibodies (bsAbs) (7), and the adoptive transfer of genetically engineered T cells expressing chimeric antigen receptors (CARs) (8). However, only a limited proportion of patients benefit from these strategies. Therefore, intense efforts are being made to improve the currently available immunotherapies and to design new strategies to strengthen anti-tumor immune responses.

## Current T Cell-Redirecting Strategies

T cell-redirecting immunotherapies are intended to specifically eliminate tumor cells by physically joining lymphocytes and cancer cells using tumor-targeted cell-cell bridging (CCB) molecules (9). CCBs can be generated using engineering approaches to manipulate the membrane of immune cells (cell surface engineering), to create artificial soluble molecules (antibody engineering) or a combination thereof (4, 5). In fact, some of these CCB-based strategies, such as membrane-anchored CARs or soluble T cell-redirecting bsAbs (T-bsAbs), are revolutionizing the treatment of B cell malignancies (10).

### CAR-Engineered T (CAR-T) Cells

**Figure 1:**
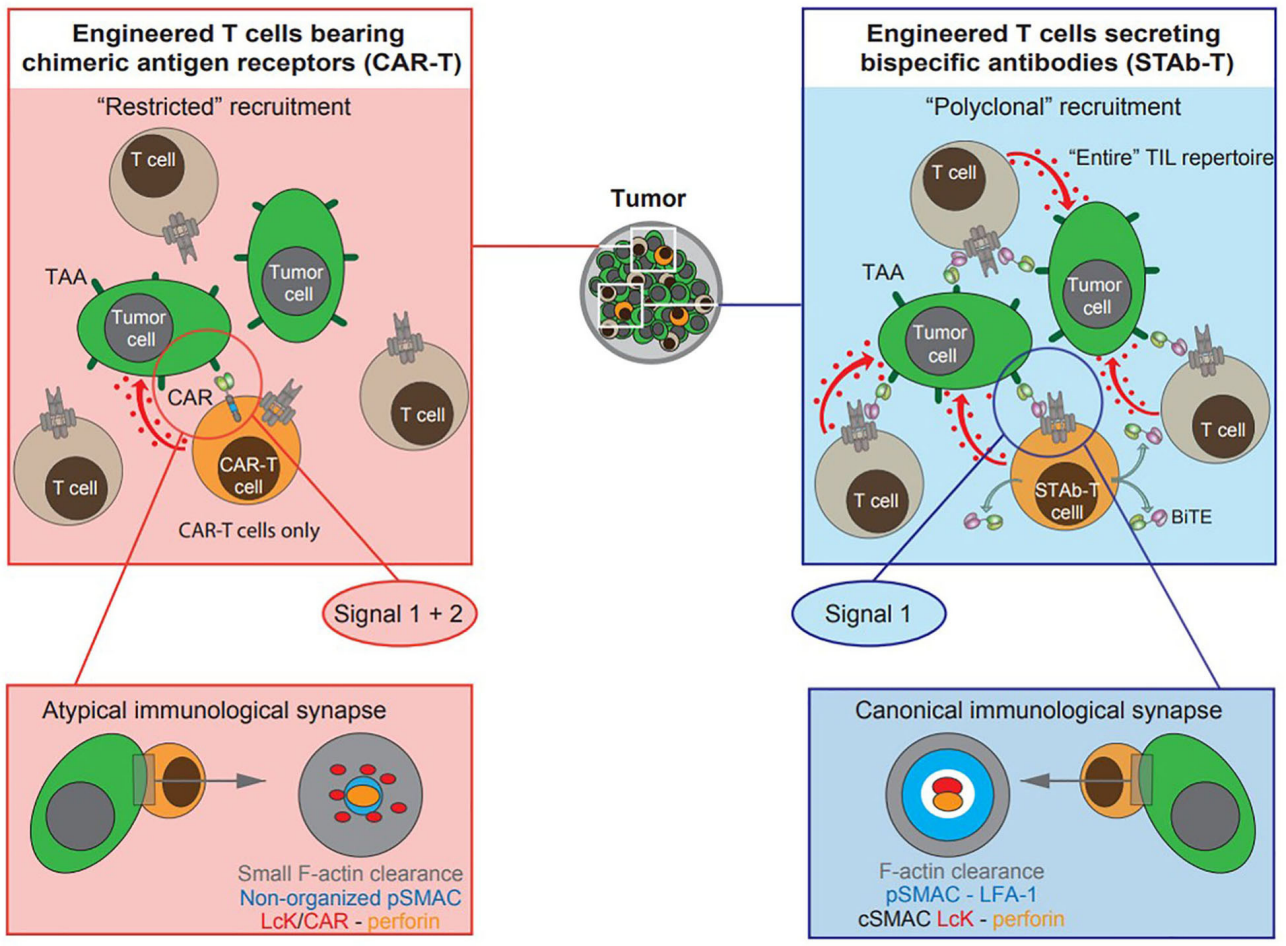
Schematic diagram depicting cell-based T cell-redirecting strategies for cancer immunotherapy. Engineered T cells (orange cells) expressing second-generation scFv-based chimeric antigen receptors (CAR-T cells), and engineered T cells secreting T cell-redirecting bispecific antibodies (STAb-T cells) in BiTE format. The tumor-associated antigen (TAA)-specific scFv is displayed in light green and the anti-CD3ζ scFv in magenta. Red arrows and dots represent delivery of the “lethal hit” to tumor cells (green cells) by CAR- or BiTE-activated T cells: engineered and/o bystander non-engineered tumor infiltrating T lymphocytes (TILs, gray cells). In engineered T cells expressing second generation CARs, a single molecular interaction provides both signals 1 and 2, whereas TAA-specific BiTEs do not provide co-stimulatory signaling to T cells. Topology observed in CAR-mediated and BiTE-mediated immunological synapse (IS): the CAR-mediated IS shows a rather disordered structure whereas the BiTE-mediated IS displays a well-organized canonical “bull's eye” structure.

CARs are synthetic receptors consisting of three domains: an antigen-binding ectodomain, the transmembrane domain, and the signaling endodomain (5). The ectodomain is usually a single-chain fragment variable (scFv) antibody, that allows the synthetic receptor to specifically recognize a user-defined cell surface TAA in an major histocompatibility complex (MHC)-independent manner, and is tethered to the transmembrane domain through the spacer or hinge region (8) (Figure 1). The third component is the endodomain, most often the CD3ζ intracellular signaling domain linked to one or more co-stimulatory domains (5, 11). First-generation CARs contain solely the intracellular signaling region of CD3ζ (12). Second-generation CARs generated by adding a co-stimulatory domain (from CD28 or CD137) in tandem with the CD3ζ chain (13) have been a major advance in CAR-T cell therapy because co-stimulation is a necessary component of physiological T cell activation, thereby improving proliferation, survival, cytokine secretion and cytotoxicity. Third-generation CARs further expanded on the second-generation by adding an additional co-stimulatory domain (14, 15).

This structure endows CAR-T cells with several valuable attributes for a T cell-redirecting strategy. As CARs are not MHC-restricted, they can be used to treat patients without regard to MHC haplotypes, and circumvent MHC down-regulation, one of the most important mechanisms of immune evasion (11). In addition, CARs provide both activating and co-stimulatory signals which are required to achieve full T cell activation (Figure 1 and Table 1) (16). The success of anti-CD19 CAR-T cells in clinical trials prompted the approval of two second generation CAR-T cells products, tisagenlecleucel (2017) and axicabtagene ciloleucel (2018), by the US FDA for the treatment of pediatric and young adult patients with relapsed or refractory B cell acute lymphoblastic leukemia (B-ALL) (17) and adult patients with relapsed or refractory large B cell lymphomas (18), respectively.

**Table 1:** Pros and cons of current T cell-redirecting strategies.

	**Adoptive cell therapies**		**Protein-based therapies**
	**CAR-T cells**	**STAb-T cells**	**Systemic administration T-bsAbs**
Active trafficking to tumor sites	✓	✓	×
Co-stimulatory signal/s	✓	×^a^/✓^b^	×
Long lifespan	✓	✓	×^c^/✓^d^
“*Off-the-shelf”* therapy	×^e^/✓^f^	×^g^/✓^h^	✓
Polyclonal recruitment of T cells	×	✓	✓
Canonical immunological synapse	×	✓	✓

a *Monocystronic approach*.

b *Bicystronic approach*.

c *Bolus therapy with small Fc-less T-bsAbs (e.g., BiTE)*.

d *Continuous intravenous infusion (CIV)/Half-extension technologies or Fc-engineered Ig “silent” T-bsAbs*.

e *Autologous CAR-T cells*.

f *“Universal” CAR-T cells*.

g *On-tumor strategy*.

h *Off-tumor strategy/”Universal” STAb-T cells*.

Nevertheless, the use of CAR-T cells presents some limitations (19), mainly severe toxicities related to a massive release of pro-inflammatory cytokines (cytokine release syndrome, CRS) and neurotoxicity (20). In addition, the majority of TAAs are also expressed on normal tissues, leading to on-target/off-tumor toxicity (19, 21). Solid tumors present additional challenges, due to the highly immunosuppressive tumor environment. Additionally, 30–60% of patients that achieve complete response, relapse after anti-CD19 CAR-T cell therapy (22).

### Bispecific Antibodies

BsAbs are artificial molecules recognizing two different epitopes either on the same or on different antigens, and by simultaneously recognizing a cell surface TAA and an activating receptor on the T cell surface (CD3e), are able to activate and redirect T effector cells to kill cancer cells in a MHC-independent manner (5, 23). In recent years a considerable number of new bsAb formats have been designed, many of which are small-sized Fc-less molecules, built by connecting scFv and/or single-variable domain/heavy chain-only (V_HH_) antibodies (23, 24). These antibodies are specifically designed to promote an efficient T cell/tumor cell synapse formation, and avoiding Fc-induced off-target toxicities (24). Among them, diabodies consist of two polypeptidic chains containing counterpaired V_H_ and V_L_ domains, connected by a short linker that prevents intramolecular pairings, resulting in the formation of dimeric molecules (55 kDa) (25). Tandem scFvs (ta-scFvs), consist of two scFvs connected by a flexible linker on a single polypeptide chain (57 kDa) (26). Those bispecific ta-scFv antibodies recognizing a tumor cell surface TAA and CD3e on T cells are so-called **bi**specific **T** cell-**e**ngagers (BiTEs) (26). The bispecific **li**ght **T**-cell **e**ngager (LiTE), consisting of a TAA-specific V_HH_ antibody fused to an anti-CD3scFv, is a recent evolution of this concept (27). The smaller size (43 kDa) and quicker diffusion of LiTE antibodies could allow them to reach tumor areas, which are inaccessible for larger bsAbs (27).

More than 30 T cell-redirecting bsAbs (T-bsAbs) have entered clinical development (28), but only one is presently in clinical use: blinatumomab, an anti-CD19 BiTE, for the treatment of relapsed/refractory B-ALL (29) and minimal residual disease-positive B-ALL (30). Despite the impressive responses observed with blinatumomab (31, 32), significant challenges still hamper the clinical application of BiTEs and similar bsAb formats. Off-target toxicities (mainly CRS and neurotoxicity), due to the expression of the targeted TAA on non-tumor cells, is a major concern for patients treated with systemically administered BiTEs (33). In addition, the short serum half-life of small-sized T-bsAbs requires continuous intravenous administration at a constant flow rate using infusion pumps (34). Another concern regarding the use of T-bsAbs is the lack of co-stimulatory signaling capacity. However, the ability of BiTEs to induce potent T cell cytotoxicity in the absence of co-stimulation has been well-documented (35). Although the reasons for this “co-stimulation independence” are not clear, it may result from the ability of Fc-less T-bsAbs to induce the formation of conventional mature immunological synapses (ISs) between T cells and tumor cells (36, 37).

## Next-Generation T Cell-Redirecting Strategies

As previously described, both CAR-T cells and systemically infused T-bsAbs have shown encouraging clinical responses but still must overcome important hurdles. In an attempt to combine the strengths of both therapies a novel strategy based on the endogenous **s**ecretion of **T**-bs**Ab**s (STAb) is being developed. We have previously classified STAb strategies as “*on-tumor*” and “*off-tumor*” depending on whether the T-bsAbs are secreted in the tumoral or peritumoral environment or from tumor-distant locations, respectively (10). The *in vivo* production of small-sized T-bsAbs by genetically modified T cells could result in effective and persistent concentrations of antibodies, compensating for their short-serum half-life (10). Moreover, this approach might circumvent problems of tumor penetration and systemic toxicity, due to tumor trafficking of adoptively transferred T cells and subsequent intratumoral secretion of T-bsAbs (Table 1) (10). In addition, *in vivo* secretion avoids potential concerns regarding the formulation and long-term storage of bsAb therapeutics, preventing aggregation and deterioration (10, 38). Finally, in the STAb-T strategy, and in contrast with CAR-T therapy, T cell recruitment is not restricted to engineered T cells, as T-bsAbs secreted in the tumor may redirect bystander non-engineered infiltrating T cells to tumor cells, leading to a significant boost in anti-tumor T cell responses (Figure 1 and Table 1) (4).

The STAb concept is now attracting attention but is not new. In 2003, a study demonstrated that human cells could be engineered to secrete a functionally active anti-CEA x anti-CD3 diabody, with ability to redirect T cell-mediated cytotoxicity against CEA-expressing tumor cells *in vitro*, and recruit bystander T cells *in vivo* to delay tumor growth (39). Moreover, anti-CEA x anti-CD3 diabody-secreting primary T cells were generated by lentiviral transduction and such STAb-T cells significantly reduced *in vivo* tumor growth in human colon cancer xenografts (40). More recently, the ability of an anti-EphA2 BiTE secreted by retrovirally transduced primary T cells demonstrated the ability of STAb-T cells to redirect the cytotoxic activity of non-transduced T cells specifically to EphA2^+^ cancer cells *in vitro* and showed potent anti-tumor activity *in vivo* (41). Likewise, systemic infusion of retrovirally transduced T cells secreting an anti-CD19 BiTE induced tumor regression of leukemia and lymphoma in preclinical models (42). Another study reported that STAb-T cells secreting an anti-CD123 BiTE redirected bystander T cell cytotoxicity against CD123^+^ acute myelod leukemia (AML) cells and induced regression of AML in xenograft models (43). Interestingly, efficient STAb-T cells have been generated not only using viral vectors, but also by RNA-transfection. In this regard, anti-CD19 STAb-T cells generated by electroporation of a messenger RNA encoding an anti-CD19 BiTE showed superior anti-tumor activities compared with RNA anti-CD19 CAR-T cells, achieving complete remission in a leukemia mouse model (44). It has been demonstrated that *in situ* secreted anti-CD19 BiTEs are loaded onto the T cell surface (42, 44). Therefore, it is tempting to speculate that the “arming of the CD3 complex” by *in vivo* secreted BiTEs in the peritumoral environment, could provide a significant therapeutic advantage over systemically administered BiTEs (e.g., blinatumomab).

Other cell types, such as mesenchymal stem cells (MSCs), and endothelial cells are suitable candidates to be engineered for “off-tumor” STAb strategies, based on the endogenous secretion of T-bsAbs from tumor-distant sites (45–47). The feasibility of *in vivo* secretion of T-bsAbs after systemic or local delivery of several types of nucleic acids or viruses has also been demonstrated (10, 48). Systemic administration of engineered mRNA (49) or minicircle DNA encoding T-bsAbs (50) induced sustained antibody secretion in mice and elimination of established human carcinoma xenografts. In another study, a single intramuscular injection of plasmid DNA induced secretion of functional T-bsAbs for 4 months and delayed cancer progression in mice (51). In addition, several types of oncolytic viruses have been armed with expression cassettes encoding T-bsAbs, to combine both direct oncolysis and T cell-mediated killing (52–55).

Nevertheless, T cells represent ideal vehicles for STAb therapy due to their capacity to migrate to tumor sites and their ability to act simultaneously as antibody factories and effectors (10). In addition, T-bsAb-mediated activation has been shown to induce an increase in transgene expression (41), which may favor the secretion of the T-bsAbs primarily at the tumor site and, consequently, reducing systemic toxicity.

## Open Questions and Future Prospects

### Co-stimulatory and Co-inhibitory Receptors

Although only extensive research and clinical trials will determine the ultimate therapeutic potential of next-generation T cell-redirecting strategies, the STAb strategy may have important conceptual advantages over the CAR strategy (10), such as the polyclonal recruitment of the entire pool of tumor infiltrating T cells, and the reduction of systemic on-target/off-tumor toxicity due to the local secretion of the T-bsAbs (Figure 1 and Table 1) (4, 5). In fact, Liu et al. have shown greater anti-leukemia activities of anti-CD19 BiTE-RNA electroporated T cells, compared to anti-CD19 CAR RNA-electroporated T cells in a Nalm6 tumor model (44). The authors highlighted the potential of anti-CD19 STAb-T cells to cure CD19^+^ neoplasia with controlled toxicities (44). By contrast, Choi et al. have reported differences between CAR-T and STAb-T cells in terms of persistence and exhaustion, supporting the notion that CAR-T cells might be superior (56). In the experimental system used, T cell activation mediated by a locally secreted anti-EGFR BiTE resulted in a progeny of phenotypically exhausted cells, with reduced proliferative capacity and persistence, compared to anti-EGFRvIII CAR-activated T cells (56). The authors suggest that these differences may be attributable to the 4-1BB co-stimulatory domain used in the CAR construct (56), although the influence of other factors, such as the different TAA targeted, their cell density, as well as the location of the epitope recognized by both anti-EGFR and anti-EGFRvIII scFvs has not been considered. The positive effects of 4-1BB-mediated co-stimulation on reducing T cell exhaustion have also been demonstrated on engineered T cells expressing a second-generation anti-CD19 CAR (BBζ) (57).

STAb-T cells have demonstrated significant anti-tumor activity in different preclinical models, without additional co-stimulation (40–42, 44, 58). However, the provision of co-stimulatory signals may be instrumental to enhance anti-tumor efficacy especially in the context of solid tumors. In fact, we have demonstrated that simultaneous secretion of an anti-CEA x anti-CD3 diabody and a tumor-specific co-stimulatory ligand comprising the extracellular portion of CD80 fused to an anti-CEA antibody (59) increased anti-tumor activity in human colon carcinoma xenografts (4). Recent studies have shown that the expression of 4-1BB and CD80 ligands on the surface of engineered T cells secreting and anti-CD19 BiTE significantly increased the antileukemia activity *in vivo* (60). Collectively, these studies showed that STAb-T cells could be easily equipped with physiological or tumor-specific co-stimulation systems using cell surface or antibody engineering strategies.

On the other hand, blockade of the PD-1/PD-L1 interaction can induce durable anti-tumor responses in a wide range of solid and hematological tumors (61). Several blocking antibodies against PD-1/PD-L1 have been approved for clinical use in humans (6), and preclinical studies have demonstrated that combining PD-1/PD-L1 axis blockade with CAR-T cells or systemically administered T-bsAb can improve anti-tumor activity (62–64). Importantly, several studies have demonstrated the therapeutic potential of engineered CAR-T cells secreting either anti-PD-1 or anti-PD-L1 blocking antibodies, and are currently being evaluated in clinical trials (65). In addition, CAR-T cells that express PD-1 dominant-negative receptors (66) or chimeric PD-1:CD28 switch-receptors (67) have been reported to increase anti-tumor effects and reduce susceptibility to tumor-induced T cell dysfunction. Finally, the rapid advancements in precision genome editing techniques, such as CRISPR-Cas9 system, has enabled to disrupt PD-1 function in CAR-T cells/T cells for cancer therapy (68, 69). All these “protective strategies” could also be easily implemented in a STAb-T cell context to improve their therapeutic potential.

### Tumor Antigen Escape

Another relevant issue in a tumor-specific T cell-redirecting context is the loss of the targeted TAA. Here, it is important to highlight that among relapsing patients treated with anti-CD19 CAR-T cells, 10–20% are CD19-negative (22), while CD19 loss is infrequent following blinatumomab therapy (7). Several mechanisms have been proposed to explain antigen loss, such as accumulation of genetic and epigenetic mutations during tumor progression and selection of antigen-negative variants due to immune pressure (22). Interestingly, trogocytosis, a process whereby lymphocytes capture fragments of the plasma membrane from antigen-presenting cells and express them on their own surface (70), has been reported to occur following CAR-T cell interaction with CD19 (71). Trogocytosis leads to reversible antigen loss that reduces both TAA density on tumor cells and CAR expression on the T cell surface, presumably as a consequence of CAR internalization. Moreover, the transfer of CD19 protein from leukemia cells to T cells promotes fratricidal T cell killing and T cell exhaustion (71). Trogocytic target acquisition seems to be a general feature of CAR-T cells, as this phenomenon has been observed with CARs targeting different antigens (71). Regarding BiTE-stimulated T cells, trogocytic mechanisms have not been reported so far, although additional studies are needed to further clarify this issue.

### Immunological Synapse

An important unresolved issue refers to structure of the IS formed by the CCB molecules in T cell redirecting strategies (Figure 1) (36). Although CAR-T cell stimulation induces an efficient microtubule organizing center and lytic granule secretion, even faster than in the canonical TCR-initiated IS, the actin cytoskeleton is not completely depleted from the center of the synapse, that exhibited a disorganized multifocal signaling cluster structure, with major differences relative to the typical TCR-initiated IS (36, 72–74). Unlike CARs, small-sized T-bsAbs are able to induce the formation of a canonical “bull's eye” IS between T lymphocytes and tumor cells (35). Indeed, BiTE-initiated IS has been found to be identical in structure and molecular composition to TCR-induced IS (37). Further studies are needed to more precisely define the impact of the topology of the IS on the functional capacity and cytotoxic potential of CAR-T and STAb-T cells (36).

### Development of Off-the-Shelf Universal Adoptive Cell Therapies

The use of allogeneic cells from healthy donors has significant advantages over autologous approaches, such as the immediate availability of cryopreserved batches and reduced cost. We have demonstrated that engineered MSCs might be incorporated into biocompatible scaffolds to secrete T-bsAbs that can act distantly at the tumor site, and can be retrieved a after a given period of time when the intended therapeutic effect has been achieved (45). Therefore, off-the-shelf stocks of gene-modified human allogeneic STAb MSCs might be easily generated and microencapsulated and implanted subcutaneously according to clinical need (75). The development of universal allogeneic CAR-T cells is an active area of research, and different strategies are being investigated to reduce the risk of graft-vs.-host disease and make cells less visible to the host immune system (76). Similar approaches could easily be implemented to the generation of universal STAb-T cells.

## Final Considerations

STAb-T cell-based strategies have demonstrated encouraging anti-tumor effects in preclinical models, but their safety needs to be further explored in controlled clinical trials. Nevertheless, the administration of CAR-T or STAb-T therapies may not necessarily be mutually exclusive and both approaches might be used sequentially or simultaneously (56). Moreover, the use of CAR-T cells and STAb-T cells targeting different TAA could be relevant to overcome antigen loss, in a fashion similar to dual-antigen CAR-T cell targeting strategies (77–80). Such a strategy might consist of the simultaneous administration of CAR and STAb-T cells or the generation of a single cell product expressing both CCBs.

## Author Contributions

BB and LA-V contributed to the conception and design of this review. BB and ÁR-F wrote the first draft of the manuscript. All authors contributed to manuscript revision, read and approved the submitted version.

## Conflict of Interest

The authors declare that the research was conducted in the absence of any commercial or financial relationships that could be construed as a potential conflict of interest.
